# Tai Chi Chuan for the Primary Prevention of Stroke in Middle-Aged and Elderly Adults: A Systematic Review

**DOI:** 10.1155/2015/742152

**Published:** 2015-02-15

**Authors:** Guohua Zheng, Maomao Huang, Feiwen Liu, Shuzhen Li, Jing Tao, Lidian Chen

**Affiliations:** ^1^College of Rehabilitation Medicine, Fujian University of Traditional Chinese Medicine, No. 1 Huatuo Road, Shangjie University Town, Fuzhou 350108, China; ^2^Fujian University of Traditional Chinese Medicine, No. 1 Huatuo Road, Shangjie University Town, Fuzhou 350108, China

## Abstract

*Background.* Stroke is a major healthcare problem with serious long-term disability and is one of the leading causes of death in the world. Prevention of stroke is considered an important strategy.* Methods. *Seven electronic databases were searched.* Results.* 36 eligible studies with a total of 2393 participants were identified. Primary outcome measures, TCC exercise combined with other intervention had a significant effect on decreasing the incidence of nonfatal stroke (*n* = 185, RR = 0.11, 95% CI 0.01 to 0.85, *P* = 0.03) and CCD (*n* = 125, RR = 0.33, 95% CI 0.11 to 0.96, *P* = 0.04). For the risk factors of stroke, pooled analysis demonstrated that TCC exercise was associated with lower body weight, BMI, FBG level, and decreasing SBP, DBP, plasma TC, and LDL-C level regardless of the intervention period less than half a year or more than one year and significantly raised HDL-C level in comparison to nonintervention. Compared with other treatments, TCC intervention on the basis of the same other treatments in patients with chronic disease also showed the beneficial effect on lowering blood pressure.* Conclusion.* The present systematic review indicates that TCC exercise is beneficially associated with the primary prevention of stroke in middle-aged and elderly adults by inversing the high risk factors of stroke.

## 1. Introduction

Although the rates of stroke mortality have declined over recent decades in most of developed countries, stroke still occupies the third commonest cause of mortality following heart disease and cancer, and resulting in around 6 million deaths annually in the world [1]. In China, approximately 2 million adults suffer a new stoke every year [2], and 6 million adults are currently standing stroke [3]. It is estimated that 700,000 people sustain a new stroke each year in the United States and on average every 40 second someone in this country has a stroke [4]. According to WHO, stroke also was one of the leading causes of adult acquiring disability and a major contributor to health-care cost worldwide. Lifetime costs per patient are estimated at between US$59,800 and US$230,000 [5]. In the UK, the direct and indirect societal costs caused by stroke are about 8.9 billion pounds a year [6]. The risk of suffering from stroke is governed by more than 100 risk factors which are classified as nonmodifiable, potentially modifiable, and modifiable factors. Prevention for stroke, in which its strategy aims are to control the risk of stoke by modifying one or more modifiable risk factors, such as physical activity level, obesity, cholesterol levels, blood pressure, smoking status, and glucose intolerance, plays a crucial role in counteracting morbidity and mortality related to stroke and is considered to be the best approach in reducing the burden of stroke [7]. It has been estimated that 50% of stroke are preventable through control of modifiable risk factors, in which exercise contributes an important part [8]. Regular exercise has favorable effects on controlling risk factors of stroke and reducing the incidence rate of a first-ever stroke [9, 10].

Tai Chi Chuan (TCC) exercise originated in China as a martial art is gentle and vigorous exercise with low impact and low-moderate intensity which involves a series of slow, continuous, and graceful body movements [11]. To date, TCC has developed into several styles which can be differentiated by the varying forms or postures, the order of the movement sequence, pace of movement, and the angle of knee flexion during the practice [12, 13]. The commonly practiced styles include “Yang,” “Chen,” “Wu,” or “Sun” styles among which the Yang style is the most popular and the Chen style is the oldest [12]. Though there are differences of posture and the position of the center of gravity, all styles incorporate slowness, rhythmic movements, relaxation, mental concentration, movement coordination, and flow into the next one with elements of meditation, body awareness, and imagery while breathing deeply [13].

TCC is a suitable exercise for people with different ages, different physical and health conditions, because it is easily accessible and of low cost, and can be easily implemented in the community setting. As an exercise for promoting health, TCC has been practiced for hundreds of years in China and is gradually acceptable in the West countries. A systematic review indicated that intensive TCC exercise shown some favorable effects on improving general cardiorespiratory fitness and its functional status, and was potentially beneficial for cardiovascular disease of elderly population [14]. A substantial amount of studies reported that TCC was efficient to control many risk factors of stroke [15–20], but there has not been a comprehensive systematic review to examine the primary preventive effect of TCC for stroke. The objective of the current study was to attempt to conduct a systematic review and meta-analysis of the existing studies on TCC exercise as an intervention for the primary prevention of stroke in middle-aged and elderly adults to draw more useful conclusions about the safety and efficacy of TCC in preventing stroke, and to offer recommendations for future research.

## 2. Methods

### 2.1. Literature Search

We searched the following electronic databases: PubMed Database, EMBASE (OVID) Database, Science Citation Index (SCI), Wanfang degree and conference papers database, China National Knowledge Infrastructure (CNKI), and Chinese Science and Technology Periodical Database (VIP) from their inception to 31 October 2013, and the Cochrane Central Register of Controlled Trials (Cochrane Library, 2013, Issue 3). The search terms included “Stroke,” “Tai Ji,” “cerebral hemorrhage,” “infarction,” “blood pressure,” “cholesterol,” and “blood sugar.” The details of search strategy are listed in Appendix A. In addition, we checked references list of reviews and retrieved articles for additional studies. No language restrictions were applied to any searches.

### 2.2. Eligibility Criteria

Randomized controlled trials (RCTs), quasirandomized controlled trials (quasi-RCTs), prospective nonrandomized controlled trials (NRCTs), self-controlled trials and first stage of cross-over trials whether published or unpublished were included. The target population was aged 30 or older with or without high risk factors of stroke. Type of TCC exercise was not limited at a frequency of at least 30 minutes per time and 3 times per week for 4 weeks. Trials with comparison of TCC exercise versus nonintervention or TCC exercise plus other treatment versus same other treatment were included, but trials with comparison of TCC exercise versus other exercise intervention or TCC exercise plus other treatment versus other exercise intervention plus same other treatment were excluded. Primary outcome measures were incidence rates of fatal or nonfatal stroke or cardia-cerebrovascular disease (CCD), and secondary outcome measures included any modification risk factor of stroke, such as blood pressure, blood lipids, fasting blood glucose (FBG).

### 2.3. Study Identification and Data Extraction

Two reviewers (HMM and LSZ) assessed the eligibility of the searched studies independently. The full-text articles that met the eligible criteria were obtained, and the relevant references were retrieved according to predefined eligibility criteria. Data concerning details of participants' characteristics, study methods, interventions, and outcomes were extracted independently by two reviewers (HMM and LSZ) through using a form based on predefined selection criteria. We resolved any disagreements of study identification and data extraction by consensus and consulted a third reviewer (ZGH) if disagreements persisted. We also contacted original author to provide additional relevant information if necessary.

### 2.4. Risk of Bias in Individual Studies

Risk of bias of the included studies was assessed using the Cochrane Collaboration's tool for assessing risk of bias by two reviewers (HMM, LFW) independently [21]. Six following criteria were applied: adequate sequence generation, concealment of allocation, blinded of primary outcomes, adequately addressed incomplete outcome data, free from selective reporting, and free of other risk of bias [21]. In addition, we assessed the baseline characteristics between the comparison groups. The disagreements between two reviewers were resolved through discussion.

### 2.5. Statistical Analysis

Data were processed in accordance with the Cochrane Handbook for Systematic Reviews of Interventions [20]. Meta-analysis was carried out using Review Manager Software 5.2 (2011, Cochrane Collaboration and Updated Software). Relative risk (RR) with 95% confidence interval (CI) was calculated for dichotomous variables. For continuous outcomes, net changes were compared a mean difference (MD) or standardized mean difference (SMD), and corresponding 95% confidence interval (CI) were calculated for each study. Heterogeneity test of each outcome was conducted using the Chi-square test with non significance (*P* > 0.05) indicating no heterogeneity among studies [22]. Degree of heterogeneity was evaluated using *I* ^2^ statistic [22]. Where there was no heterogeneity, a fixed-effect model was performed in meta-analysis, otherwise random effects model was used. If substantial heterogeneity was detected, the review authors looked for possible explanations, and considered to use the following options: provide a narrative overview, not aggregate the studies at all, or use a random-effect model with appropriate cautious interpretation. Subgroup analysis or sensitivity analysis was applied to explore the cause of heterogeneity among studies.

## 3. Results

### 3.1. Study Identification

**Figure 1:**
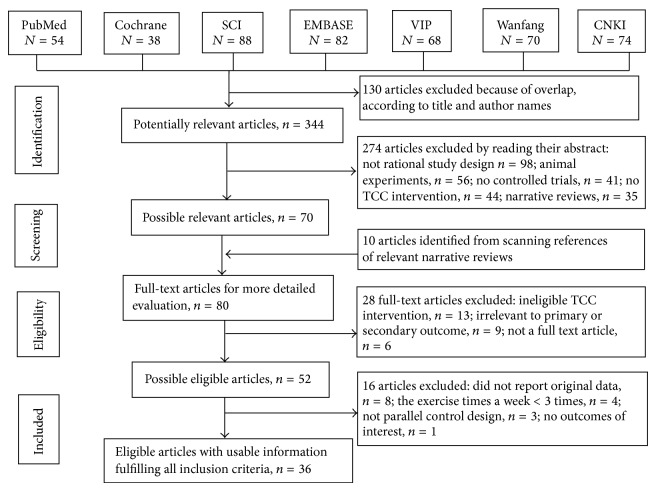
Flow diagram for search and selection of the included studies.

Figure 1 summarizes the flow of the literature search and selection process. A total of 474 records were identified from the relevant databases, and 130 duplicate records were excluded. Among the 344 potential articles, 274 were excluded by reading the title and abstract. 80 full-text articles including ten of which was identified from the reference lists were evaluated for their eligibility. 44 articles were further excluded because they did not meet the inclusion criteria. A list of excluded studies can be found in Appendix B. Finally, 36 studies with a total of 2393 participants were eligible to be included in this systematic review [23–58].

### 3.2. Characteristics of Included Studies

Characteristics of the methods, participants, intervention, comparison group and outcome measures of each included studies in this review were shown in Table 1. Among the 36 included studies, only one [36] was conducted in Mexico and others conducted in China. 2 of 36 articles were reported in English, and the remaining reported in Chinese. Participants in the included studies involved in healthy individuals or patients with chronic diseases, and their ages ranged from 30 to 82 years. 22 trials [23–44] compared TCC with nonintervention. The remaining trials were designed comparing TCC plus conventional treatment with same conventional treatment. In these trials, frequency of TCC intervention was at least 3 times one week, more than 30 minutes per time with at least 1 month duration. Two studies reported the primary outcomes including the incidence of fatal, nonfatal stroke and cardia-cerebrovascular disease [48, 58], and others mainly focused on the risk factors of stroke such as body weight, blood pressure, blood lipids, or blood glucose. No studies reported the adverse events.

### 3.3. Methodological Quality of Included Studies

**Figure 2:**
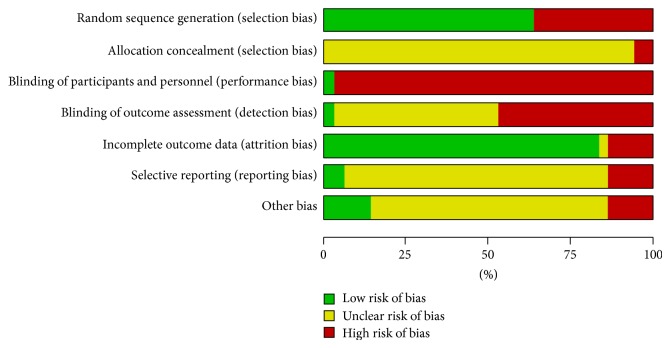
“Risk of bias” graph: review authors' judgments about each risk of bias item presented as percentages across all included studies.

Details of the risk of bias assessed for each of the included studies are summarized in Figure 2 and Appendix C. Of the 36 included studies, 23 studies reported randomization allocation, but only 4 studies [27, 29, 48, 52] described the method of randomization by using random number tables or stratified random distribution. No study reported the allocation concealment. Only one trial [37] clearly described the outcome assessors blinded. Five studies [28, 31, 33, 36, 58] reported numbers of participants who dropped out, and one of five [33] performed the intention to treat analysis and another [58] reported follow-up period. As a whole, 8 out of 36 studies (22.2%) were judged as at high risk of bias because one or more main aspects of the bias assessment was labeled high, and other studies were judged as at unclear risk of bias. We tried to contact the original authors but most of them had no response.

### 3.4. Measures of Effect

#### 3.4.1. Incidence of Fatal, Nonfatal Stroke, Cardia-Cerebrovascular Disease and Cardiac Failure

Two studies with 185 elders reported the primary outcomes including incidence of fatal and nonfatal stroke [48, 58], the incidence of CCD [45], and the incidence of cardiac failure [58]. Compared to other intervention, TCC exercise combined with other intervention had a significant effect to decrease the incidence of nonfatal stroke (*n* = 185, RR = 0.11, 95% CI 0.01 to 0.85, *P* = 0.03) and CCD (*n* = 125, RR = 0.33, 95% CI 0.11 to 0.96, *P* = 0.04), but the significant effects were not observed on the incidence of fatal stroke (*n* = 185, RR = 0.33, 95% CI 0.05 to 2.05, *P* = 0.23) and cardiac failure (*n* = 60, RR = 0.17, 95% CI 0.02 to 1.30, *P* = 0.09) (Table 2).

#### 3.4.2. Body Weight, BMI, WHR, Waistline, Hip Circumference

Meta-analysis showed the significant differences between TCC intervention and nonintervention on decreasing body weight (*n* = 241, MD = −3.21 kg, 95% CI −5.18 to −1.24, *P* = 0.001, *I* ^2^ = 0%), and body mass index (BMI) (*n* = 381, MD = −1.01 kg/m^2^, 95% CI −1.34 to −0.69, *P* < 0.00001, *I* ^2^ = 3%) in healthy participants. One study [35] with 25 healthy participants also showed TCC intervention was efficient to reduce waist-hip ratio (WHR) in comparison to nonintervention (MD = −2.40, 95% CI −4.53 to −0.27, *P* = 0.03) (Table 3).

Another study [58] in 125 healthy participants measured body weight, waistline, and hip circumference after two years intervention, and results showed that TCC exercise plus health education were better than health education alone on reducing body weight (MD = −4.30 kg, 95% CI −7.29 to −1.31, *P* = 0.005), waistline (MD = −7.00 cm, 95% CI −10.1 to −3.90, *P* < 0.00001), and hip circumference (MD = −4.60 cm, 95% CI −6.91 to −2.29, *P* < 0.00001) (Table 3).

#### 3.4.3. Blood Pressure

12 studies with 832 healthy participants comparing TCC intervention with nonintervention and 10 studies mainly involving in patients with chronic diseases comparing TCC intervention plus other intervention with same other intervention (conventional treatment or health education) reported the effect of TCC intervention for blood pressure. Compared to nonintervention, TCC intervention with intervention period for more than one year had significant reduction on systolic blood pressure (SBP) (*n* = 198, MD = −9.58 mmHg, 95% CI −14.54 to −4.61, *P* = 0.0002, *I* ^2^ = 0%), or diastolic blood pressure (DBP) (*n* = 198, MD = −4.00 mmHg, 95% CI −7.44 to −0.56, *P* = 0.02, *I* ^2^ = 11%). The pooled results of TCC intervention for less than half a year also showed the significant difference between TCC intervention and nonintervention on SBP (*n* = 634, MD = −11.98 mmHg, 95% CI −17.50 to −6.47, *P* < 0.0001) and DBP (*n* = 634, MD = −6.11 mmHg, 95% CI −9.92 to −2.29, *P* = 0.002) (Table 4). But their heterogeneities among studies also were substantive (for both SBP and DBP *I* ^2^ = 90%), sensitivity analysis, for SBP, after excluding one study with a low quality score [38] and the self-controlled trial [33], the effect size remained statistical significance with acceptable heterogeneity (*n* = 378, MD = −15.15 mmHg, 95% CI −19.60 to −10.71, *P* < 0.00001, *I* ^2^ = 68%); for DBP, after excluding one study [28], the effect size was reduced but remained statistical significance with moderate heterogeneity (*n* = 547, MD −4.06 mmHg, 95% CI −6.12 to −1.99, *P* = 0.0001, *I* ^2^ = 63%).

Table 4 also showed that TCC intervention plus other intervention for patients with chronic diseases was far superior to same other intervention used alone regarding blood pressure reduction. There were significant differences between comparison groups after intervention regardless of less than half a year (SBP: *n* = 406, MD = −14.21 mmHg, 95% CI −17.54 to −10.88, *P* < 0.00001, random model; DBP: *n* = 406, MD = −7.08 mmHg, 95% CI −9.06 to −5.09, *P* < 0.00001, random model), or more than one year (SBP: *n* = 624, MD = −8.29 mmHg, 95% CI −9.63 to −6.95, *P* < 0.00001, random model; DBP: *n* = 624, MD = −4.56 mmHg, 95% CI −6.45 to −2.67, *P* < 0.00001, random model), and the heterogeneity among studies was also expressed a acceptable range from *I* ^2^ = 0% to *I* ^2^ = 60%.

#### 3.4.4. Blood Lipid Levels (TC, TG, HDL-C, and LDL-C)

There were 14 studies mainly involving in healthy participants comparing TCC intervention with nonintervention and 2 studies with 98 patients with chronic diseases comparing TCC intervention plus conventional treatment with same conventional treatment. They reported the effects of TCC for blood lipid levels including total cholesterol (TC), triglycerides (TG), high-density lipoprotein cholesterol (HDL-C) or low-density lipoprotein cholesterol (LDL-C).

For TC, TCC intervention showed a reduction of TC level at intervention period less than half a year (*n* = 340, SMD = −0.87, 95% CI −1.66 to −0.08, *P* = 0.03, *I* ^2^ = 90%) or more than one year (*n* = 298, SMD = −0.32, 95% CI −0.63 to −0.02, *P* = 0.04, *I* ^2^ = 40%) in comparison to nonintervention (Table 5). Since there was significant heterogeneity in the comparison of subgroup with less than half a year intervention period (*I* ^2^ = 90%), we examined the data carefully and found one study [23] deviated from the others. We performed a sensitive analysis through removing this study, and got a similar result with no heterogeneity (*n* = 280, SMD −0.43, 95% CI −0.66 to −0.19, *P* = 0.0005, *I* ^2^ = 0%). There was one study [45] with 60 patients with chronic disease reported TCC intervention plus conventional treatment versus same conventional treatment, and result showed a significant reduction of TC level (MD = −1.76 mmol/L, 95% CI −2.02 to −1.50, *P* < 0.00001) (Table 5).

Subgroup analysis according to different TCC intervention period found significant reduction of TG level at TCC intervention period for less than half a year (*n* = 340, SMD = −0.58, 95% CI −1.03 to −0.14, *P* = 0.01, *I* ^2^ = 72%), or more than one year (*n* = 298, SMD = −0.67, 95% CI −0.90 to −0.43, *P* < 0.00001, *I* ^2^ = 0%) (Table 5). One study [45] comparing TCC intervention plus conventional treatment with same conventional treatment reported there was no significant difference on serum TG level in patients with chronic disease (*n* = 60, MD = −0.05 mmol/L, 95% CI −0.27 to 0.17, *P* = 0.66) (Table 5).

Compared to nonintervention, TCC intervention showed a increase of HDL-C level regardless of intervention period for less than half a year (*n* = 340, SMD = 0.77, 95% CI 0.01 to 1.53, *P* = 0.05, *I* ^2^ = 90%) or more than one year (*n* = 335, SMD = 0.88, 95% CI 0.44 to 1.32, *P* < 0.0001, *I* ^2^ = 73%) (Table 5). But the heterogeneity in subgroup analysis of TCC intervention period more than one year were substantive with *I* ^2^ value being 90%, and the subtotal meat-analysis showed no significant difference (*n* = 280, SMD = 0.40, 95% CI −0.07 to 0.86, *P* = 0.09, *I* ^2^ = 67%) after sensitivity analysis was performed through excluded one study [23]. One study [45] with 60 patients with chronic disease reported TCC intervention plus conventional treatment versus same conventional treatment on HDL-C level, and result showed a significant increase of HDL-C level (MD = 0.20 mmol/L, 95% CI 0.16 to 0.24, *P* < 0.00001) (Table 5).

Subgroup analysis showed TCC intervention in comparison to nonintervention could significantly reduce the LDL-C level at intervention period less than half a year (*n* = 340, SMD = −0.95, 95% CI −1.64 to −0.26, *P* = 0.007), or more than one year (*n* = 371, SMD = −0.93, 95% CI −1.72 to −0.15, *P* = 0.02). However, their heterogeneities of both comparisons also were substantive with *I* ^2^ value being 87% and 91%, respectively (Table 5). Sensitivity analysis showed TCC intervention remained significant reduction of LDL-C level at intervention period less than half a year (*n* = 280, SMD = −0.58, 95% CI −0.82 to −0.33, *P* = 0.0002, *I* ^2^ = 29%) excluded one study [23], but at intervention period more than one year, after excluding any study, the effect size was remained statistically significant but still indicated high heterogeneity among studies. Two studies [45, 49] with 98 patients with chronic disease reported TCC intervention plus conventional treatment versus same conventional treatment on LDL-C level, and pooled analysis was not performed because of the high heterogeneity (*I* ^2^ = 96%) existed between the two studies. One study [45] reported a significant difference between comparison groups (*n* = 60, SMD = −0.54, 95% CI −0.73 to −0.35, *P* < 0.00001), and another study [49] reported no significant difference (*n* = 38, SMD = −0.05, 95% CI −0.13 to 0.03, *P* = 0.22) (Table 5).

#### 3.4.5. FBG, PBG, FPI

6 studies mainly involving in healthy participants comparing TCC intervention with nonintervention and 2 studies with 64 patients with chronic disease comparing TCC intervention plus conventional treatment with same conventional treatment reported FBG level. Compared to nonintervention, TCC intervention showed a significant reduction of FBG level (*n* = 230, SMD = −0.93, 95% CI −1.42 to −0.43, *P* = 0.0003, *I* ^2^ = 67%) (Table 6). Compared with same conventional treatment used alone in patients with chronic disease, TCC combined with conventional treatment did not show a significant difference on FBG level (*n* = 64, MD = 0.60 mmol/L, 95% CI −0.94 to 2.14, *P* = 0.45, *I* ^2^ = 73%) (Table 6).

One study [30] with 32 healthy participants reported the effect of TCC intervention on postprandial two-hour blood glucose (PBG) in comparison to nonintervention, and results showed a significant reduction of PBG level (MD = −1.40 mmol/L, 95% CI −1.64 to −1.16, *P* < 0.0001) (Table 6).

There were two studies [29, 31] that reported the effect of TCC intervention on fasting plasma insulin (FPI) in comparison to nonintervention, meat-analysis was not used for significant difference because of a high heterogeneity (*I* ^2^ = 91%), and in this comparison, TCC intervention showed a significant reduction of FPI level (*n* = 40, MD = −6.80 U/L, 95% CI −10.42 to −3.48, *P* < 0.0001) in one study [29] and no significant difference (*n* = 19, MD = 7.99 U/L, 95% CI −1.41 to 17.39, *P* = 0.1) in another study [31] (Table 6). One study [56] with 40 patients with chronic disease reported TCC intervention plus conventional treatment versus same conventional treatment, and result showed no significant difference of FPI level (MD = −0.10 U/L, 95% CI −0.3 to 0.10, *P* = 0.32) (Table 6).

#### 3.4.6. Adverse Effects

None of included studies reported adverse events.

## 4. Discussion

A comprehensive search was conducted through major electronic databases for interventions involving in TCC exercise. Reference lists of systematic review were also screened when necessary. All screening of eligible studies, data extraction and analysis were carried out independently by two review authors. Our decision to restrict the investigated interventions to comparing TCC exercise or/plus conventional treatment with nonintervention/or same conventional treatment in this review avoided the potential confounding effects of other behavioral interventions on the outcomes, such as those involving other exercises, different dietary interventions or interventions that focused on weight loss. In this systematic review, 36 studies accounting for 2393 participants (1094 healthy adults and 1299 patients with chronic diseases) were identified. Only 4 out of 23 included RCTs reported definitive randomization, 2 studies were self-controlled trials, and the remaining trials were controlled trials without randomization. We attempted to contact authors by telephone or e-mail for further information. But most replies were unsatisfactory and did not resolve our questions even no response. Therefore, as a whole, the majority of the included studies belonged to low methodological quality. Of all studies, 22 studies were designed to compare TCC with nonintervention [23–44]. The remaining studies were designed to compare TCC plus conventional treatment with the same conventional treatment.

In the primary outcomes, two studies with 185 participants assessed incidence of nonfatal, fatal stroke, cardia-cerebrovascular disease (CCD), or cardiac failure after five years' follow-up period, their results showed TCC exercise had a beneficial effects on preventing the incidence of nonfatal stroke and CCD. However, because of insufficient data, we cannot make any conclusion about the effects of TCC preventing the occurrence of stroke directly, more trials which investigated the effects of TCC on the clinic outcomes of stroke with long follow-up period are need. Nonetheless, in this review we also examined secondary outcomes mainly including the risk factors of stroke such as body weight, blood pressure, lipid levels and blood glucose and so on. In the present review, compared with nonintervention, TCC intervention remarkably lowered body weight, BMI, SBP, DBP and FBG on healthy adults; It also significantly decreased plasma TC, TG, and LDL-C level, and raised HDL-C level. Compared with other treatment (conventional treatment or health education), TCC intervention on the basis of same other treatment could significantly lower SBP, DBP and plasma LDL-C level in patients with chronic diseases. The results showed the comprehensive controlled effect of TCC intervention on risk factors of stroke and indicated that it might be effective in preventing stroke in healthy adults or patients with chronic diseases. Because small reductions in risk factors of stoke may lead to larger reductions in stoke incidence throughout a whole population [59]. Nevertheless, concluding that TCC have definitively preventive effects on stoke would be premature because most of the studies were of low methodological quality with obvious design shortcomings such as inadequate concealment, no blinding, and incomplete outcomes data point to the possibility of bias. Additionally, clinical heterogeneity was apparent because different style of TCC was used.

Up to present, some systematic reviews have looked at TCC for cancer, balance, sleep quality, osteoarthritis, fibromyalgia and rheumatoid arthritis [60–65], or have look at TCC for the rehabilitation of stroke [66]. We did not find other systematic review had been carried out to examine the effects of TCC for the primary prevention of stroke in adults. Other reviews have examined the effects of TCC for blood pressure, blood lipid, and blood glucose on healthy old population or patients with chronic disease [19, 20, 67, 68]. Their results indicated limited evidence for the effectiveness of TCC on blood pressure and blood lipid other than blood glucose in the elderly. One review investigated the effect of TCC for the primary prevention of CVD, and found some suggestions of beneficial effects of TCC on CVD risk factors, but this review was based on only 13 trials with a small sample sizes and the conclusion cannot be drawn because of inconsistency across all included trials [69]. In present review, we analyzed 36 studies accounting for 2393 participants, the results shown that TCC exercise had the significant benefit in modifying the risk factors of stoke in middle-aged and elderly adults such as body weight, blood pressure, lipid levels and blood glucose. Researches have demonstrated stroke can be prevented through controlling its modifiable risk factors, and small reductions of risk factors may lead to larger decrease in the incidence of stroke throughout a whole population [8–10]. Accordingly, we thought that TCC exercise might be beneficial for the primary prevention of stroke by modifying the risk factors. Furthermore few studies in present review also found that TCC with a long term training significantly decreased the incidence of nonfatal stroke [48, 58]. Therefore, there was some suggestion of beneficial effects of TCC exercise on the primary prevention of stroke but large long-term trials are needed to confirm this evidence.

Several limitations were identified in this review. The key limitation was quality of the included studies. Mostly included studies were of low quality due to no definite on random sequence generation, allocation concealment, and the blinding outcome assessors. The potential bias might erode the reliability of conclusions of this review. Secondly, the direct effect of TCC preventing the occurrence of stroke cannot be assessed due to no enough follow-up period performed in the included studies. Thirdly, funnel plot analysis could not be conducted due to the insufficient number of studies included for any outcomes in this review, however, majority of included studies published in China. Therefore the possible publication bias is inevitable. In addition, the variations on the characteristics of participants and intervention period indicated some clinical heterogeneity among the included trials.

In this current review, few studies evaluate TCC for the primary preventive effect of stroke by examining the direct outcomes, but the results suggest that TCC exercise may be effective in modifying the risk factors of stroke. Due to the limitation of methodological quality and the limited evidence available, currently no conclusion can be drawn as to the effectiveness of TCC exercise for the primary prevention of stroke. Large prospective long-term trials with rigorous design are needed before TCC exercise can be recommended as a population-based intervention to prevent the incidence of stroke.

## Figures and Tables

**Figure 3:**
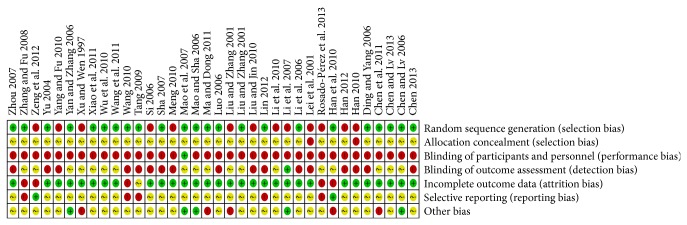
“Risk of bias” summary: review authors' judgments about each risk of bias each included study.

**Table 1:** The Characteristics of Included Studies in This Systematic Review.

Author, year	Methods	Participants total (T/C)	Mean (SD)/range age; subjects	Intervention		Frequency and duration of TCC intervention	Outcomes
				Treatment	Control		
Ding and Yang 2006 [23]	RCT	60 (30/30)	mean 65/66 (T/C) years; Hyperlipidemia patients	TCC	Nonintervention	50 minutes per time and 5 times a week for 20 weeks	TC, TG, HDL-C, LDL-C
Liu and Zhang 2001 [24]	RCT	86 (44/42)	mean age: 64.8 years; Healthy elders	TCC	Nonintervention	above 60 minutes per time and twice a day for more than 48 weeks	BMI, TC, TG, HDL-C, LDL-C
Ma and Dong 2011 [25]	RCT	30 (15/15)	middle-aged and elderly adults Hyperlipidemia patients	TCC	Nonintervention	60 minutes every day for 24 weeks	TC, TG, HDL-C, LDL-C
Mao et al. 2007 [26]	RCT	80 (40/40)	mean 63 years; Healthy elderly women	TCC	Nonintervention	45 to 60 minutes per time and 3 times a day for 48 weeks	TC, TG, HDL-C, LDL-C
Sha 2007 [27]	RCT	40 (20/20)	mean 64.5/63.4 (M/W) years; Healthy elders	TCC	Nonintervention	60 to 120 minutes per time and 5 to 7 times a week for 72 weeks	TC, TG, HDL-C, LDL-C
Wang 2010 [28]	RCT	85 (40/45)	Mean 56.46/56.34 (T/C) years; Healthy elderly women	TCC	Nonintervention	30 minutes per time and 4 times a week for 18 weeks	SBP, DBP
Wu et al. 2010 [29]	RCT	40 (20/20)	mean 51.3/52.4 (T/C) years; Healthy elders	TCC	Nonintervention	above 60 minutes per time and above 3 times a week for 24 weeks	SBP, DBP, FBG, FPI
Yan and Zhang 2006 [30]	RCT	32 (16/16)	mean 50.5/50.8 (T/C) years; Healthy elders	TCC	Nonintervention	above 30 minutes per time, once a day and 5 to 7 times a week for 48 weeks	TC, TG, FBG, PBG
Zhang and Fu 2008 [31]	RCT	20 (10/10)	mean 57.4 ± 6.2 years; Type 2 diabetes patients	TCC	Nonintervention	60 minutes a day, and 5 times a week for 14 weeks	TC, TG, HDL-C, LDL-C, FBG, FPI
Zhou 2007 [32]	RCT	120 (60/60)	mean 52.3/53.4 (T/C) years; Type 1 EH patients	TCC	Nonintervention	60 minutes a day for 12 weeks	SBP, DBP, TC, TG, HDL-C, LDL-C
Han 2010 [33]	SC	50	mean 60.5/61.7 (M/W) years; Healthy elders	TCC	Nonintervention	60 minutes per time and mean 4 times per week for 24 weeks	SBP, DBP
Lei et al. 2001 [34]	SC	39	mean 61.87 years; Healthy elders	TCC	Nonintervention	30 minutes per time and once or twice a day for 48 weeks	SBP, DBP
Han 2012 [35]	NRCT	25 (15/10)	mean 61.2/57.1 (T/C) years; Healthy elders	TCC	Nonintervention	60 minutes per time and 3 times a week for 12 weeks.	Weight, BMI, WHR, SBP, DBP, TC, TG, HDL-C, LDL-C, FBG
Rosado-Pérez et al. 2013 [36]	NRCT	25 (15/10)	mean 66.4/66.7 (T/C) years; Healthy elders	TCC	Nonintervention	60 minutes every day for 24 weeks	BMI, SBP, DBP, TC, TG, HDL-C, LDL-C, FBG
Li et al. 2007 [37]	NRCT	32 (16/16)	mean 58 years; Healthy elders	TCC	Nonintervention	45 minutes per time, 5 times a day at learning phase of 4 weeks and 60 minutes per time, 5 times a day at practice period of 20 weeks.	TC, TG, HDL-C, LDL-C
Li et al. 2010 [38]	NRCT	156 (79/77)	mean 67.44/69.40 (T/C) years; Healthy elders	TCC	Nonintervention	above 30 minutes per time and above 3 times a day for more than 24 weeks	Weight, BMI, SBP, DBP
Liu and Jin 2010 [39]	NRCT	20 (10/10)	range from 53 to 68 years; Healthy elders	TCC	Nonintervention	60 minutes per time and 4 times per week for 8 weeks	SBP, DBP
Liu and Zhang 2001 [40]	NRCT	69 (36/33)	range from 60 to 71 years; Healthy elders	TCC	Nonintervention	for 2 years	HDL-C, LDL-C
Meng 2010 [41]	NRCT	36 (25/11)	mean 49.4/47.09 (T/C) years; Healthy elders	TCC	Nonintervention	above 14 hours a week and mean time of exercise is 14.7 ± 8.6 months	LDL-C
Si 2006 [42]	NRCT	60 (30/30)	mean 55.3/56.6 (T/C) years; Healthy elders	TCC	Nonintervention	40 to 60 minutes per time and above 4 times a week for more than 2 years	BMI, SBP, DBP, TC, TG, HDL-C, LDL-C, FBG
Xu and Wen 1997 [43]	NRCT	34 (18/16)	mean 66.7/64.6 (T/C) years; Healthy elders	TCC	Nonintervention	30 minutes per time and twice a day for 4 weeks	SBP, DBP
Yang and Fu 2010 [44]	NRCT	60 (30/30)	range from 50 to 70 years; Healthy elderly men	TCC	Nonintervention	90 minutes per time and more than 4 times per week for 48 weeks	Weight, SBP, DBP
Chen 2013 [45]	RCT	60 (32/28)	mean 69.3/68.7 (T/C) years; CHD patients	TCC plus conventional treatment	The same conventional treatment	60 minutes per time and 4 times a week for 12 weeks	TC, TG, HDL-C, LDL-C
Chen and Lv 2006 [46]	RCT	40 (20/20)	mean 64.3/60 (T/C) years; EH patients	TCC plus conventional treatment	The same conventional treatment	40 minutes every day for 9 weeks	SBP, DBP
Chen and Lv 2013 [47]	RCT	68 (50/18)	range from 30 to 82 years; EH patients	TCC plus conventional treatment	same conventional treatment	30 minutes per time and 6 times a week for 12 weeks	SBP, DBP
Han et al. 2010 [48]	RCT	60 (30/30)	mean 62.12 years; EH patients	TCC plus routine treatment	The same routine treatment	45 to 60 minutes per time and 1 to 2 times a week for 48 weeks	SBP, DBP, incidence of fatal, non-fatal stroke and cardiac failure
Li et al. 2006 [49]	RCT	38 (20/18)	mean 66/67 (T/C) years; CHD patients	TCC plus conventional treatment	The same conventional treatment	40 minutes per time, twice a day and above 6 times a week for 24 weeks	LDL-C
Lin 2012 [50]	RCT	60 (30/30)	mean 60 years; Type 2 diabetes patients	TCC plus conventional treatment	The same conventional treatment	90 minutes every day for 12 weeks	SBP, DBP
Luo 2006 [51]	RCT	84 (44/40)	mean 44.74/44.84 (T/C) years; EH patients	TCC plus conventional treatment	The same conventional treatment	45 minutes per time and once a day for 24 weeks	SBP, DBP
Mao and Sha 2006 [52]	RCT	62 (51/11)	mean 62.2/63.3 (T/C) years; EH patients	TCC plus conventional treatment	The same conventional treatment	60 minutes per time and 6 time a week for 8 weeks	SBP, DBP
Tang 2009 [53]	RCT	32 (16/16)	mean 63.65/62.79 (T/C) years; EH patients	TCC plus conventional treatment	The same conventional treatment	30 to 60 minutes per time and 3 to 5 times a week for 24 weeks	SBP, DBP
Wang et al. 2011 [54]	RCT	60 (30/30)	range from 50 to 70 years; Type 2 and 3 EH patients	TCC plus conventional treatment	The same conventional treatment	120 minutes per time and 5 times a week for 16 weeks	SBP, DBP
Xiao et al. 2011 [55]	RCT	24 (12/12)	mean 55 ± 4.12 years; Type II diabetes patients	TCC plus conventional treatment	The same conventional treatment	60 minutes per time, once a day and 6 times a week for 24 weeks	FBG
Yu 2004 [56]	RCT	40 (20/20)	mean 50/49 (T/C) years; Type II diabetes and EH patients	TCC plus conventional treatment	The same conventional treatment	60 minutes per time and once a day for 12 weeks	FBG, FPI
Chen et al. 2011 [57]	RCT	441 (238/203)	range from 35 to 75 years; EH patients	TCC plus conventional treatment plus health education	The same conventional treatment plus same health education	60 minutes per time and 5 times a week for 2 years	SBP, DBP
Zeng et al. 2012 [58]	NRCT	125 (63/62)	range from 45 to 70 years; Healthy elders	TCC plus health education	The same health education	30 to 40 minutes per time, and no less than 3 times a week for 2 years	Weight, waistline, hip circumference, SBP, DBP, incidence of fatal, non-fatal stroke and CCD

BMI: body mass index; CCD: cardia-cerebrovascular disease; CHD: coronary heart disease; DBP: diastolic blood pressure; EH: essential hypertension; FBG: fasting blood glucose; FPI: fasting plasma insulin; HDL: high-density lipoprotein; LDL: low-density lipoprotein; M/W: man/woman; NRCT: prospective nonrandomized controlled trial; PBG: postprandial two-hour blood glucose; RCT: randomized controlled trial; SC: self-controlled trial; SBP: systolic blood pressure; TCC: Tai Chi Chuan; T/C: treatment/control; TC: total cholesterol; TG: triglycerides; WHR: waist-hip rate.

**Table 2:** The effect of TCC on incidence of fatal stroke, non-fatal stroke, cardia-cerebrovascular disease, and cardiac failure.

Outcome or subgroup title	Number of studies	Number of participants	Effect size; risk ratio (M-H, fixed, 95% CI)	*P* value	*I* ^2^	P_Heterogeneity_
Tai Chi Chuan plus other intervention versus same other intervention						
Incidence of nonfatal stroke	2	185	0.11 [0.01, 0.85]	0.03	0*℅*	0.65
Incidence of fatal stroke	2	185	0.33 [0.05, 2.05]	0.23	0*℅*	0.64
Incidence of CCD	1	125	0.33 [0.11, 0.96]	0.04		
Incidence of cardiac failure	1	60	0.17 [0.02, 1.30]	0.09		

CCD: cardia-cerebrovascular disease.

**Table 3:** The effect of TCC on body weight, body mass index, waist-hip ratio, waistline, and hip circumference.

Outcome or subgroup title	Number of studies	Number of participants	Effect size; mean difference (IV, fixed, 95% CI)	*P* value	*I* ^2^	P_Heterogeneity_
Tai Chi Chuan versus nonintervention						
Body weight (kg)	3	241	−3.21 [−5.18, −1.24]	0.001	0*℅*	0.58
BMI (kg/m^2^)	5	381	−1.01 [−1.34, −0.69]	<0.00001	3*℅*	0.39
WHR	1	25	−2.40 [−4.53, −0.27]	0.03		

Tai Chi Chuan plus health education versus same health education						
Body weight (kg)	1	125	−4.30 [−7.29, −1.31]	0.005		
Waistline (cm)	1	125	−7.00 [−10.10, −3.90]	<0.00001		
Hip circumference (cm)	1	125	−4.60 [−6.91, −2.29]	<0.00001		

BMI: body mass index; WHR: waist-hip ratio.

**Table 4:** The effect of TCC on blood pressure.

Outcome or subgroup title	Number of studies	Number of participants	Effect size; std. mean difference (IV, random, 95% CI)	*P* value	*I* ^2^	P_Heterogeneity_
Tai Chi Chuan versus nonintervention for blood pressure						
SBP (mmHg)						
Intervention for less than half a year	9	634	−11.98 [−17.50, −6.47]	<0.0001	90*℅*	<0.00001
Intervention for more than 1 year	3	198	−9.58 [−14.54, −4.61]	0.0002	0*℅*	0.45
DBP (mmHg)						
Intervention for less than half a year	9	634	−6.11 [−9.92, −2.29]	0.002	90*℅*	<0.00001
Intervention for more than 1 year	3	198	−4.00 [−7.44, −0.56]	0.02	11*℅*	0.32

Tai Chi Chuan plus other interventions versus same other interventions						
SBP (mmHg)						
Intervention for less than half a year	7	406	−14.21 [−17.54, −10.88]	<0.00001	43*℅*	0.11
Intervention for more than 1 year	3	624	−8.29 [−9.63, −6.95]	<0.00001	0*℅*	0.92
DBP (mmHg)						
Intervention for less than half an year	7	406	−7.08 [−9.06, −5.09]	<0.00001	47*℅*	0.08
Intervention for more than 1 year	3	624	−4.56 [−6.45, −2.67]	0.0002	38*℅*	0.20

SBP: systolic blood pressure; DBP: diastolic blood pressure.

**Table 5:** The effect of TCC on blood lipids.

Outcome or subgroup title	Number of studies	Number of participants	Effect size; std. mean difference (IV, random, 95% CI)	*P* value	*I* ^2^	P_Heterogeneity_
Tai Chi Chuan versus nonintervention						
TC						
Intervention for less than half a year	7	340	−0.87 [−1.66, −0.08]	0.03	90*℅*	<0.00001
Intervention for more than 1 year	5	298	−0.32 [−0.63, −0.02]	0.04	40*℅*	0.15
TG						
Intervention for less than half a year	7	340	−0.58 [−1.03, −0.14]	0.01	72*℅*	0.002
Intervention for more than 1 year	5	298	−0.67 [−0.90, −0.43]	<0.00001	0*℅*	0.82
HDL-C						
Intervention for less than half an year	7	340	0.77 [0.01, 1.53]	0.05	90*℅*	<0.00001
Intervention for more than 1 year	5	335	0.88 [0.44, 1.32]	<0.0001	73*℅*	0.006
LDL-C						
Intervention for less than half an year	7	340	−0.95 [−1.64, −0.26]	0.007	87*℅*	<0.00001
Intervention for more than 1 year	6	371	−0.93 [−1.72, −0.15]	0.02	91*℅*	<0.00001

Tai Chi Chuan plus conventional treatment versus same conventional treatment						
TC (mmol/L)	1	60	−1.76 [−2.02, −1.50]			<0.00001
TG (mmol/L)	1	60	−0.05 [−0.27, 0.17]			0.66
HDL-C (mmol/L)	1	60	0.20 [0.16, 0.24]			<0.00001
LDL-C (mmol/L)						
Chen 2013 [45]	1	60	−0.54 [−0.73, −0.35]			<0.00001
Li et al. 2006 [49]	1	38	−0.05 [−0.13, 0.03]			0.22

TC: total cholesterol; TG: triglycerides; HDL-C: high-density lipoprotein cholesterol; LDL-C: low-density lipoprotein cholesterol.

**Table 6:** The effect of TCC on fasting blood glucose, postprandial two-hour blood glucose, and fasting plasma insulin.

Outcome or subgroup title	Number of studies	Number of participants	Effect size; mean difference (IV, fixed, 95% CI)	*P* value	*I* ^2^	P_Heterogeneity_
Tai Chi Chuan versus non-intervention						
FBG	6	230	−0.93 [−1.42, −0.43]^♦^	0.0003	67*℅*	0.01
PBG (mmol/L)	1	32	−1.40 [−1.64, −1.16]	<0.00001		
FPI						
Wu et al. 2010 (U/L) [29]	1	40	−6.80 [−10.12, −3.48]	<0.0001		
Zhang and Fu 2008 (pM) [31]	1	19	7.99 [−1.41, 17.39]	0.1		

Tai Chi Chuan plus conventional treatment versus same conventional treatment						
FBG (mmol/L)	2	64	0.60 [−0.94, 2.14]	0.45	73*℅*	0.05
FPI (U/L)	1	40	−0.10 [−0.30, 0.10]	0.32		

^♦^Std: mean difference (IV, random, 95% CI).

FBG: fasting blood glucose; PBG: postprandial two-hour blood glucose; FPI: fasting plasma insulin.

**Table 7:** Characteristics of excluded studies [ordered by study ID].

Study	Reason for exclusion
Bi and Chen, 2005^[1]^	No outcomes of interest
Chang et al., 2013^[2]^	Frequency of intervention is less than 3 times a week
Channer et al., 1996^[3]^	No data for extraction
Chang et al., 2013^[4]^	No data for extraction
Fu and Guo, 2013^[5]^	No data for extraction
Rosado-Perez et al., 2012^[6]^	No data for extraction
Lam et al., 2008^[7]^	Frequency of intervention is less than 3 times a week
Motivala et al., 2006^[8]^	Not parallel control design
Nguyen and Kruse, 2010^[9]^	Frequency of intervention is less than 3 times a week
Thomas et al., 2005^[10]^	No data for extraction
Thornton and Tang, 2004^[11]^	No data for extraction
Wang, 2010^[12]^	No data for extraction
Wang et al., 2004^[13]^	Not parallel control design
Wolf et al., 2003^[14]^	Not parallel control design
Wolf et al., 2006^[15]^	Frequency of intervention is less than 3 times a week
Zhang and Tan, 2006^[16]^	No data for extraction

References to studies excluded from this review:

^[1]^Y. Bi and W. H. Chen, “The effects of Tai Chi exercise on blood rheology in patients with hypertension,” *Chinese Journal of Sports Medicine*, vol. 24, pp. 606–607, 2005.

^[2]^R. Y. Chang, M. Koo, C. K. Chen, Y. C. Lu and Y. F. Lin, “Effects of habitual T'ai Chi exercise on adiponectin, glucose homeostasis, lipid profile, and atherosclerotic burden in individuals with cardiovascular risk factors,” *Journal of Alternative and Complementary Medicine*, vol. 19, pp. 697–703, 2013.

^[3]^K. S. Channer, D. Barrow, R. Barrow, M. Osborne, and G. Ives, “Changes in haemodynamic parameters following Tai Chi Chuan and aerobic exercise in patients recovering from acute myocardial infarction,” *The Fellowship of Postgraduate Medicine*, vol. 72, pp. 349–351, 1996.

^[4]^M.-Y. Chang, S.-C. J. Yeh, M.-C. Chu et al., “Associations between Tai Chi Chung Program, Anxiety, and Cardiovascular Risk Factors,” *Am J Health Promot*, vol. 28, pp. 16–22, 2013.

^[5]^X. Fu and J. Guo, “The study of the influence of Tai Chi exercise on physical fitness and its composition of the middle-aged and eldly,” *Journal of Gansu Normal Colleges,* vol. 14, pp. 73–76, 2009.

^[6]^J. Rosado-Perez, E. Santiago-Osorio, R. Ortiz et al., “Tai Chi diminishes oxidative stress in Mexican older adults,” *Journal of Nutrition, Health and Aging,* vol. 16, pp. 642–646, 2012.

^[7]^P. Lam, S. M. Dennis, T. H. Diamond et al., “Improving glycemic and BP control in type 2 diabetes: The effectiveness of Tai Chi,” *Australian Family Physician*, vol. 37, pp. 884–887, 2008.

^[8]^S. J. Motivala, J. Sollers, J. Thayer et al., “Tai Chi Chih acutely decreases sympathetic nervous system activity in older adults,” *Journals of Gerontology: Series A Biological Sciences and Medical Sciences*, vol. 61, pp. 1177–1180, 2006.

^[9]^M. H. Nguyen and R. Kruse, “The effects of Tai Chi training on physical fitness, perceived health, and blood pressure in elderly Vietnamese,” *Open Access Journal of Sports Medicine,* vol. 2012, pp. 7–16, 2012.

^[10]^G. N. Thomas, W. L. Hong Athena, B. Tomlinson et al., “Effects of Tai Chi and resistance training on cardiovascular risk factors in elderly Chinese subjects: a 12-month longitudinal, randomized, controlled intervention study,” *Clin Endocrinol (Oxf),* vol. 63, pp. 663–669, 2005.

^[11]^E. W. Thornton, K. S. Sykes, and W. K. Tang, “Health benefits of Tai Chi exercise: Improved balance and blood pressure in middle-aged women,” *Health Promotion International,* vol. 19, pp. 33–38, 2004.

^[12]^G. J. Wang, “The effects of Tai Chi exercise on cardiopulmonary function in the elderly,” *Chinese Journal of Gerontology,* p. l2, 2010.

^[13]^Y. G. Wang, G. F. Lv, and Y. B. Ren, “The effects of exercise therapy on type 2 diabetes in the middle-aged and elderly,” *Chinese Journal of Sports Medicine,* vol. 23, pp. 679–681, 2004.

^[14]^S. L. Wolf, X. Barnhart Huimnan, Kutner, G. Nancy et al., “Selected as the Best Paper in the 1990s: Reducing Frailty and Falls in Older Persons: An Investigation of Tai Chi and Computerized Balance Training,” *Journal of the American Geriatrics Society,* vol. 51, pp. 1794–1803, 2003.

^[15]^S. L. Wolf, M. O'Grady, K. A. Easley et al., “The influence of intense Tai Chi training on physical performance and hemodynamic outcomes in transitionally frail, older adults,” *Journals of Gerontology: Series A Biological Sciences and Medical Sciences,* vol. 61, pp. 184–189, 2006.

^[16]^T. M. Zhang and Y. M. Tan, “The effects of Tai Chi on fitness in middle-aged and elderly women comparing with the young,” *Chinese Journal of Clinical Rehabilitation*, vol. 10, pp. 76–78, 2006.

**Table 8:** Checklist of the PRISMA statement on systematic review.

Section/topic	Item	Checklist item	Reported on page
Title			
Title	1	Identify the report as a systematic review, meta-analysis, or both.	Title (Page 1)
Abstract			
Structured summary	2	Provide a structured summary including, as applicable: background; objectives; data sources; study eligibility criteria, participants, and interventions; study appraisal and synthesis methods; results; limitations; conclusions and implications of key findings; systematic review registration number.	Abstract (Page 1-2)
Introduction			
Rationale	3	Describe the rationale for the review in the context of what is already known.	Introduction (Page 2-3)

Title			
Objectives	4	Provide an explicit statement of questions being addressed with reference to participants, interventions, comparisons, outcomes, and study design (PICOS).	Methods
Methods			
Protocol and registration	5	Indicate if a review protocol exists, if and where it can be accessed (e.g., Web address), and, if available, provide registration information including registration number.	N/A
Eligibility criteria	6	Specify study characteristics (e.g., PICOS, length of follow-up) and report characteristics (e.g., years considered, language, publication status) used as criteria for eligibility, giving rationale.	Methods
Information sources	7	Describe all information sources (e.g., databases with dates of coverage, contact with study authors to identify additional studies) in the search and date last searched.	Methods (Page 3)
Search	8	Present full electronic search strategy for at least one database, including any limits used, such that it could be repeated.	Appendix 1
Study selection	9	State the process for selecting studies (i.e., screening, eligibility, included in systematic review, and, if applicable, included in the meta-analysis).	Methods (Page 4)
Data collection process	10	Describe method of data extraction from reports (e.g., piloted forms, independently, in duplicate) and any processes for obtaining and confirming data from investigators.	Methods (Page 4)
Data items	11	List and define all variables for which data were sought (e.g., PICOS, funding sources) and any assumptions and simplifications made.	Methods (Page 4)
Risk of bias in individual studies	12	Describe methods used for assessing risk of bias of individual studies (including specification of whether this was done at the study or outcome level), and how this information is to be used in any data synthesis.	Methods (Page 4)
Summary measures	13	State the principal summary measures (e.g., risk ratio, difference in means).	Methods (Page 4)
Synthesis of results	14	Describe the methods of handling data and combining results of studies, if done, including measures of consistency (e.g., *I* ^2^) for each meta-analysis.	Methods (Page 4)
Risk of bias across studies	15	Specify any assessment of risk of bias that may affect the cumulative evidence (e.g., publication bias, selective reporting within studies).	N/A
Additional analyses	16	Describe methods of additional analyses (e.g., sensitivity or subgroup analyses, metaregression), if done, indicating which were prespecified.	Methods (Page 4)
Results			
Study selection	17	Give numbers of studies screened, assessed for eligibility, and included in the review, with reasons for exclusions at each stage, ideally with a flow diagram.	Figure 1
Title			
Study characteristics	18	For each study, present characteristics for which data were extracted (e.g., study size, PICOS, follow-up period) and provide the citations.	Table 1
Risk of bias within studies	19	Present data on risk of bias of each study and, if available, any outcome-level assessment (see Item 12).	Figure 2
Results of individual studies	20	For all outcomes considered (benefits or harms), present, for each study: (a) simple summary data for each intervention group and (b) effect estimates and confidence intervals, ideally with a forest plot.	Tables 2–6
Synthesis of results	21	Present results of each meta-analysis done, including confidence intervals and measures of consistency.	N/A
Risk of bias across studies	22	Present results of any assessment of risk of bias across studies (see Item 15).	N/A
Additional analysis	23	Give results of additional analyses, if done (e.g., sensitivity or subgroup analyses, metaregression [see Item 16]).	Results (Page 8–10)
Discussion			
Summary of evidence	24	Summarize the main findings including the strength of evidence for each main outcome; consider their relevance to key groups (e.g., health care providers, users, and policy makers).	Discussion (Page 13)
Limitations	25	Discuss limitations at study and outcome level (e.g., risk of bias), and at review level (e.g., incomplete retrieval of identified research, reporting bias).	Discussion (Page 13)
Conclusions	26	Provide a general interpretation of the results in the context of other evidence, and implications for future research.	Discussion (Page 13-14)
Funding			
Funding	27	Describe sources of funding for the systematic review and other support (e.g., supply of data); role of funders for the systematic review.	Acknowledgments (Page 14)

## Appendices

### A. Search Strategies

#### A.1. Science Citation Index (SCI)

#1 TS: (stroke)
#2 TS: (“Cerebral hemorrhage”)
#3 TS: (Infarction)
#4 TS: (Hemiplegia)
#5 TS: (“Blood pressure”)
#6 TS: (“Systolic blood pressure”)
#7 TS: (“Diastolic blood pressure”)
#8 TS: (Cholesterol)
#9 TS: (Triglycerides)
#10 TS: (“lipoproteins, ldl”)
#11 TS: (“lipoproteins, hdl”)
#12 TS: (“Blood sugar”)
#13 #1–#12/OR
#14 TS: (“Tai ji”)
#15 TS: (Taiji)
#16 TS: (“Tai-ji”)
#17 TS: (“Tai Chi”)
#18 TS: (“Chi, Tai”)
#19 TS: (“Tai Ji Quan”)
#20 TS: (“Ji Quan, Tai”)
#21 TS: (“Quan, Tai Ji”)
#22 TS: (Taijiquan)
#23 TS: (“T'ai Chi”)
#24 TS: (“Tai Chi Chuan”)
#25 #14–#24/OR
#26 TS: (control)
#27 TS: (Comparison)
#28 TS: (“Controlled trial”)
#29 #26 OR #27 OR #28
#29 #13 AND #25 AND #29.

#### A.2. PubMed

#1 stroke [MeSH Terms]
#2 Cerebral hemorrhage [MeSH Terms]
#3 Infarction [MeSH Terms]
#4 Hemiplegia [MeSH Terms]
#5 Blood pressure [MeSH Terms]
#6 “Systolic blood pressure” [ALL]
#7 “Diastolic blood pressure”[ALL]
#8 Cholesterol [MeSH Terms]
#9 Triglycerides [MeSH Terms]
#10 lipoproteins, ldl [MeSH Terms]
#11 lipoproteins, hdl [MeSH Terms]
#12 Blood sugar [MeSH Terms]
#13 #1–#12/OR
#14 Tai ji [MeSH Terms]
#15 “Tai-ji” [ALL]
#16 “Tai Chi” [ALL]
#17 “Chi, Tai” [ALL]
#18 “Tai Ji Quan” [ALL]
#19 “Ji Quan, Tai” [ALL]
#20 “Quan, Tai Ji” [ALL]
#21 Taiji [ALL]
#22 Taijiquan [ALL]
#23 “T'ai Chi” [ALL]
#24 “Tai Chi Chuan” [ALL]
#25 #14–#24/OR
#26 control [ALL]
#27 Comparison [ALL]
#28 “Controlled trial” [ALL]
#29 #26 OR #27 OR #28
#30 #13 AND #25 AND #29.

#### A.3. The Cochrane Library

#1 MeSH descriptor: [Stroke] expllode all trees
#2 stroke or strokes
#3 cerebrovasc^*^
#4 cerebral next vascular
#5 apoplexy
#6 brain near/2 accident^*^
#7 brain^*^ near/2 infarct^*^
#8 cerebral near/2 infarct^*^
#9 lacunar near/2 infarct^*^
#10 MeSH descriptor: [Hypertension] explode all trees
#11 hypertensi^*^
#12 peripheral next arter^*^ next disease^*^
#13 high near/2 blood next pressure
#14 increased near/2 blood next pressure
#15 elevated near/2 blood next pressure
#16 MeSH descriptor: [Hyperlipidemias] explode all trees
#17 hyperlipid^*^
#18 hyperlip?emia^*^
#19 hypercholesterol^*^
#20 hypercholester?emia^*^
#21 hyperlipoprotein?emia^*^
#22 hypertriglycerid?emia^*^
#23 MeSH descriptor: [Cholesterol] explode all trees
#24 cholesterol
#25 MeSH descriptor: [Blood Pressure] this term only
#26 “blood pressure”
#27 #1–#26/or
#28 MeSH descriptor: [Tai ji] explode all trees
#29 “Tai-ji”
#30 “Tai Chi”
#31 “Chi, Tai”
#32 “Tai Ji Quan”
#33 “Ji Quan, Tai”
#34 “Quan, Tai Ji”
#35 Taiji
#36 Taijiquan
#37 “T'ai Chi”
#38 “Tai Chi Chuan”
#39 #28–#38/or
#40 control
#41 Comparison
#42 “Controlled trial”
#43 #40 or #41 or #42
#44 #27 and #39 and #42.

#### A.4. Embase Ovid

#1 exp Stroke/
#2 (stroke or stokes).tw.
#3 cerebrovasc^*^.tw.
#4 “cerebral vascular”.tw.
#5 apoplexy.tw.
#6 (brain adj2 accident^*^).tw.
#7 ((brain^*^ or cerebral or lacunar) adj2 infarct^*^).tw.
#8 exp Hypertension/
#9 hypertensi^*^.tw.
#10 “peripheral arter^*^disease^*^”.tw.
#11 ((high or increased or elevated) adj2 blood pressure).tw.
#12 exp Hyperlipidemias/
#13 hyperlipid^*^.tw.
#14 hyperlip?emia^*^.tw.
#15 hypercholesterol^*^.tw.
#16 hypercholester?emia^*^.tw.
#17 hyperlipoprotein?emia^*^.tw.
#18 hypertriglycerid?emia^*^.tw.
#19 exp Arteriosclerosis/
#20 exp Cholesterol/
#21 cholesterol.tw.
#22 “coronary risk factor^*^”.tw.
#23 Blood Pressure/
#24 “blood pressure”.tw.
#25 #1–#24/or
#26 exp Tai ji/
#27 “Tai-ji”.tw.
#28 “Tai Chi”.tw.
#29 “Chi, Tai”.tw.
#30 “Tai Ji Quan”.tw.
#31 “Ji Quan, Tai”.tw.
#33 “Quan, Tai Ji”.tw.
#34 Taiji.tw.
#35 Taijiquan.tw.
#36 “T'ai Chi”.tw.
#37 “Tai Chi Chuan”.tw.
#38 #26–#37/or
#39 control.tw.
#40 Comparison.tw.
#41 “Controlled trial”.tw.
#42 #39 or #40 or #41
#43 #25 and #38 and #42.

### B. Characteristics of Excluded Studies

See Table 7.

### C. “Risk of Bias” Summary

See Figure 3.

### D. Check list of the PRISMA Statement on Systematic Review

See Table 8.

## Abbreviations and Acronyms

BMI: body mass index
CCD: cardia-cerebrovascular disease
CVD: cardio vascular disorder
DBP: diastolic blood pressure
EH: essential hypertension
FBG: fasting blood glucose
FPI: fasting plasma insulin
HDL-C: high-density lipoprotein cholesterol
LDL-C: low-density lipoprotein cholesterol
NRCT: prospective nonrandomized controlled trial
PBF: postprandial two-hour blood glucose
RCT: randomized controlled trial
SBP: systolic blood pressure
TC: total cholesterol
TCC: Tai Chi Chuan
TG: triglycerides
WHR: waist-hip rate.
